# Microbial biofortification of fermented foods: a review of probiotic-mediated nutrient enhancement

**DOI:** 10.3389/fnut.2026.1754233

**Published:** 2026-02-12

**Authors:** Fahad Saad Alhodieb

**Affiliations:** Department of Basic Health Sciences, College of Applied Medical Sciences, Qassim University, Buraydah, Saudi Arabia

**Keywords:** fermentation, probiotics, vitamin biosynthesis, food matrices, nutrient availability, microbial strains

## Abstract

Microbial biofortification via probiotic fermentation is a unique solution to reducing micronutrient deficiencies worldwide and it is a sustainable approach to prevention and German fermentation is widely applicable for plant-based diets as these micronutrients, such as B12 and K, are hardly present. Fermentative microbes such as Lactobacillus, Bifidobacterium, Propionibacterium synthesis of the vitamins like folate, riboflavin, vitamin K. They also facilitate the accessibility of minerals and increase the quality of proteins in many foods. This process not only enhances vitamins and minerals as a result of antinutrient such as phytate breakdown, but also bioactive peptides and short-chain fatty acids are produced. These are beneficial compounds for gut health and are helpful to the health of the immune system. Studies in labs, animals, and humans indicate that consumption of biofortified fermented foods increases micronutrient levels, promotes gut microbial balance, and increases immunity. In order to exploit this approach to its fullest potential, there are hurdles to overcome, ensuring that the strain remains viable, enhancing product taste, and overcoming regulatory hurdles. Future advances will require engineering strains of probiotics to produce even greater amounts of vitamins and implementing personalized microbiome information, as well as their public health interventions, in resource-limited situations.

## 1 Introduction

Microbial biofortification is a sustainable approach to enhancing the nutritional value of foods using beneficial microbes that are responsible for the production of vitamins and nutrients during fermentation of the food. Probiotic and fermentative strains are able to produce some micronutrients such as folate (vitamin B_9_), riboflavin (vitamin B_2_), cobalamin (vitamin B_12_) and menaquinones (vitamin K). This sets a transformation of rather common foods into products rich in nutrients (1, 2). The technique is of particular value in plant-based diets, in which these vitamins are often low and deficiency is widespread in many parts of the world. By calling upon microbe natural metabolism in the fermentation process, foods are enriched with increased vitamin content, more easily absorbed minerals, a higher level of proteins for digestion and bioactive molecules useful to human health (3).

**Figure 1:**
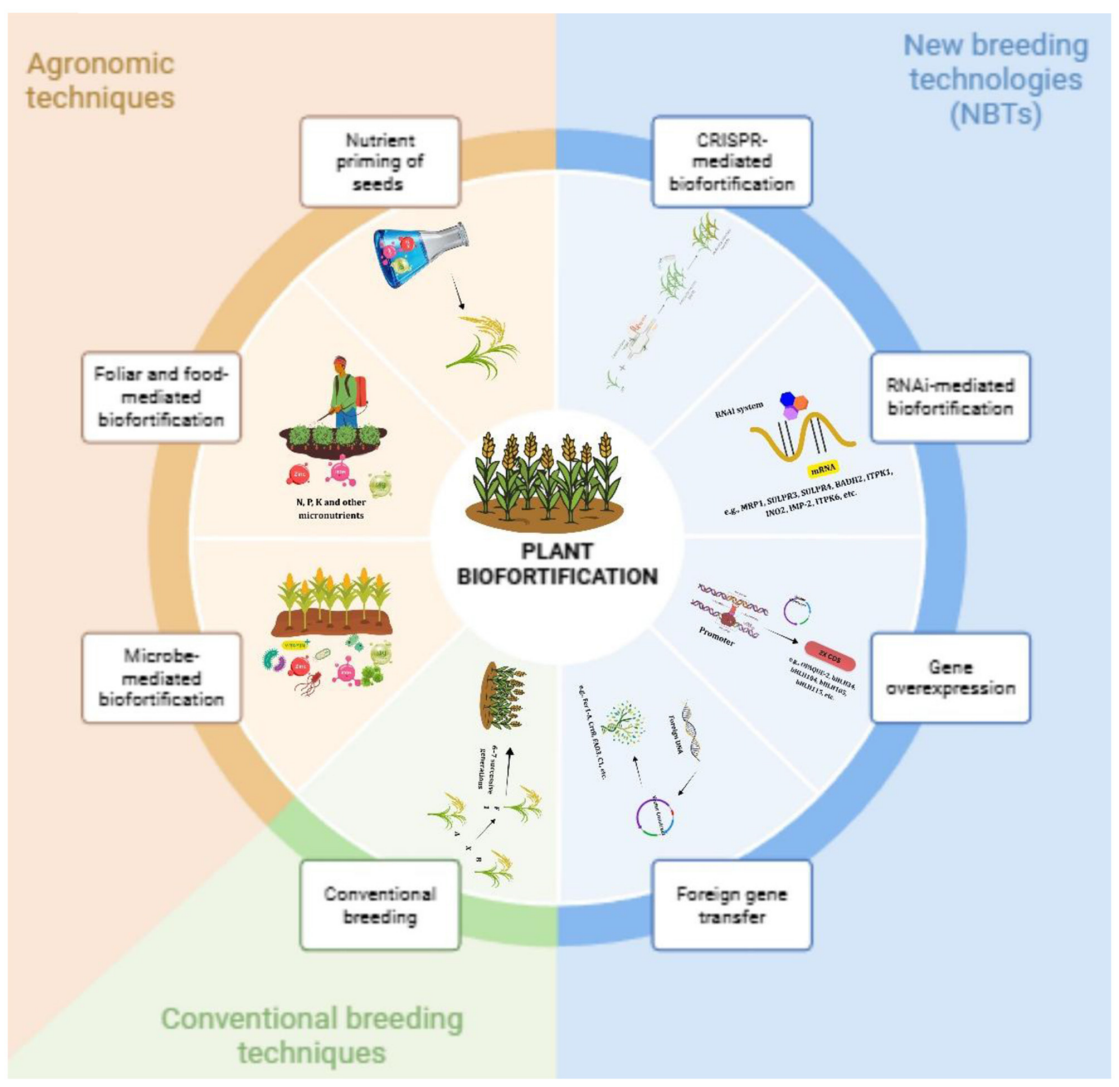
Methods of biofortification—These methods are foliar sprays, soil applications, and mineral fertilization through irrigation with the aim of increasing micronutrient uptake and deposition in the plant edible parts.

Fermented foods are a major feature of world nutrition as they are staple foods in many areas of the world. Fermentation helps extend the shelf life by improving product stability, safety, and taste of the food and provides nutritional value. Across the world, large amounts of micronutrients are supplied by fermented dairy products (yogurt, kefir, cheese), cereals (sourdough bread, dosa), legumes (tempeh, miso), and vegetables (kimchi, sauerkraut) to millions of people. Starter cultures, or natural microbes, break down raw ingredients using enzymes, which increase vitamin production, eliminate anti-nutrients (such as phytates), increase mineral solubility (Figure 1) (2, 4). For example, fermented cereals can have riboflavin contents of 2.5–5 mg/kg and have had contents of 150–340 μg/100 g of folate, much higher than the content of unfermented cereals. Soy products that are fermented with *Propionibacterium freudenreichii* can contain 0.95–3.2 μg/100 grams of vitamin B12. Considered one of the best varieties is *Bacillus subtilis* natto, which produces up to 50–100 μg/100 grams of soybean consumers Vitamin K_2_, vitally important for the health of bones and the heart. These benefits demonstrate the ability of fermented foods to fill micronutrient gaps, particularly in populations with limited access to nutrients by eating mostly plant foods (3, 5, 6).

Probiotics do more than just reside in fermented foods, they produce active vitamins this way and alter the nutritional profile of the food with the help of complex biochemistry. Strains of *Lactobacillus, Bifidobacterium, Propionibacterium* and *Streptococcus thermophilus* are highly renowned sources of B-group vitamins (i.e., riboflavin, folate and occasionally cobalamin) (4, 7). The synthesis of vitamin, probiotics have the effect to break down phytates which frees up the iron, zinc and calcium that are otherwise trapped into the plant cells. Their proteolytic activities enhance protein digestibility and provide needed lysine and other important amino acids to consume for those populations that may depend on mostly plant protein. Probiotics also help to support the gut health by balancing the microbiota and strengthening the intestinal barrier which increases the absorption and effectiveness of nutrients. Using probiotics for food production and as supplements are a natural and consumer friendly approach for food fortification (8, 9).

Despite promising advances in the use of probiotics to add nutrients to fermented foods, a number of research gaps make it difficult for probiotic use to become widespread. Strains like *Propionibacterium freudenreichii* and various lactic acid bacteria have been able to synthesize vitamins (vitamins B_2_, B_9_, B_12_, and K_2_) in food products, such as cheese, natto, and plant-based beverages (10). However, evidence that these vitamins are absorbed and have an impact is inconsistent, because most studies are small or short term and rarely are large and long term randomized controlled trials. Production also depends on the specific strain, fermentation conditions, food matrix and available precursors and there are hardly studies on the influence of individual differences such as the gut microbiota, genetic or health background, rendering personalized applications difficult. Additionally, the ongoing viability of probiotics is difficult during the large-scale production process, during storage and digestion (pH, oxygen, and bile) and competition from other microbes. Safety data on risks such as biogenic amines and infections, especially in vulnerable people, is also poor (11, 12).

New research is looking toward multi-omics techniques (e.g., genomics and metabolomics) for new vitamin-producing strains and for optimized mixtures of microbes and prebiotics such as inulin which can enhance stability and bioactive content. Precision fermentation with next generation probiotics, immobilization methods and sustainable ingredients (such as food by-products) are scalable solutions to adding nutrients. Longitudinal clinical trials are required to ensure benefit on immune function, metabolic health and nutritional security, however. Clear rules on approving the strains and health claims along with consumer education on the importance of the food matrix will help to get these products to market. These advances make enhancement of microorganisms with nutrients a sustainable alternative to a synthetic becoming (13, 14).

The review intends to provide an in-depth synthesis of the knowledge on microbial biofortification. It emphasizes metabolic pathways and genes involved in the production of vitamins by probiotic strains, the health benefits and effectiveness in practical use of these vitamins, and the real dictums of “bringing findings from living laboratories to a commercial product and public health intervention.” The functions of microbes in vitamin production the article covers strain specific capacity for production of folate, riboflavin, vitamin B_12_, and vitamin K, considers how the production pathways in microbe are regulated and how they interact in microbial communities, and reviews the evidence from lab, animal and human studies, on the impact on nutrient status, gut health and immunity, of microbially produced vitamins. It also covers technological barriers, sensory barriers, and regulatory barriers, and also challenges in the development of new products and acceptance by consumers. Future directions presented in the review include genetic engineering of probiotic strains for enhanced vitamin production, personalization of nutrition through individual microbiome specific investigation, and implementation of these approaches in household structures of nutrition programs in the developing world. The interdisciplinary approach combines microbiology, nutrition and food technology to develop innovative and sustainable solutions to help reduce micronutrient deficiency around the world (1, 15).

## 2 Mechanisms of nutrient enhancement by probiotic microorganisms

**Figure 2:**
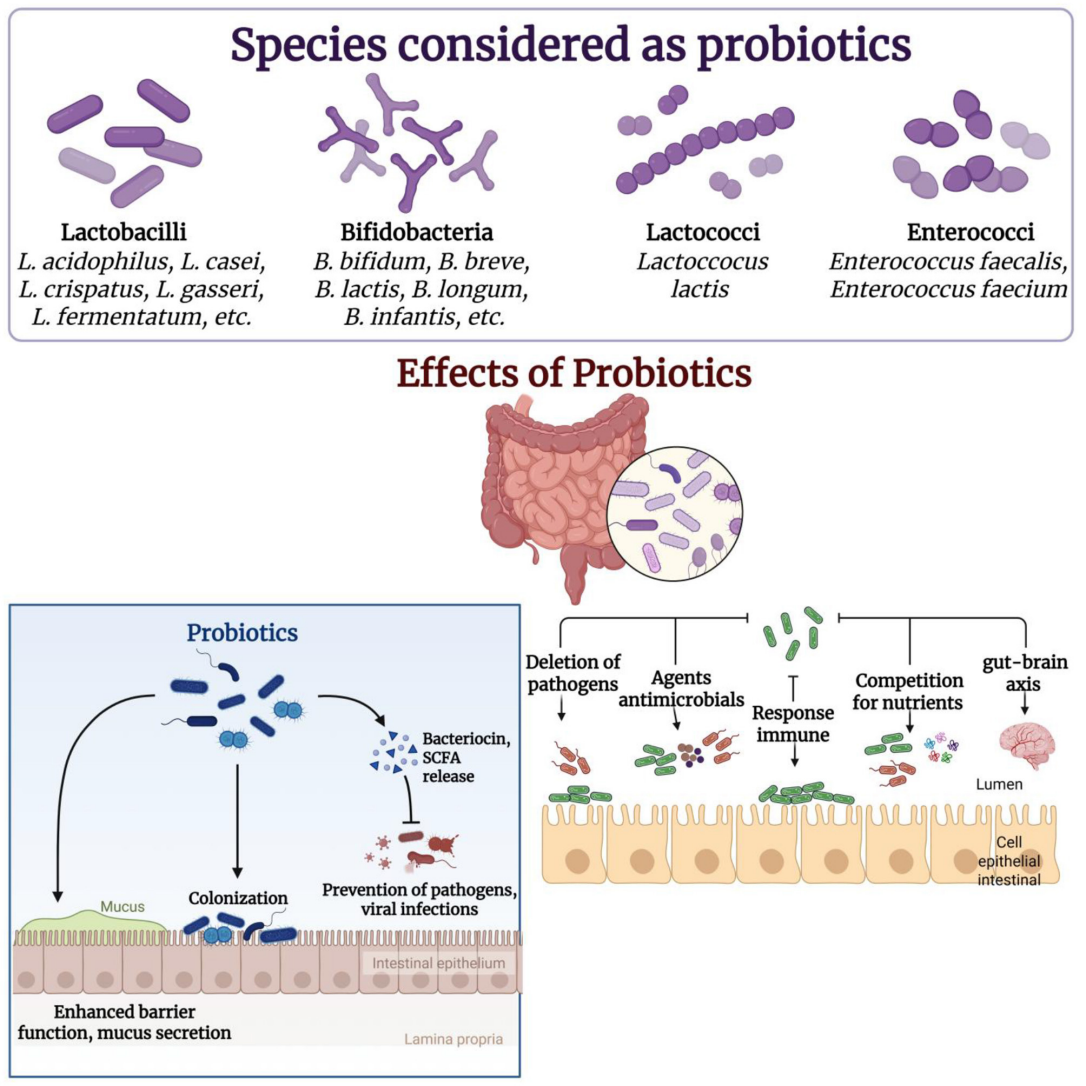
Probiotics and its mechanism of action. Created with BioRender.com.

Probiotic microorganisms enhance the nutritional value of fermented foods through a number of biochemical and physiological mechanisms (Figure 2). One of the important mechanisms is the endogenously production of vitamins in the fermentation. Some strains of probiotics, particularly lactic acid bacteria, such as *Lactobacillus plantarum, Lactobacillus reuteri* and bifidobacteria strain, contain the enzymatic pathway that is required to synthesize water-soluble B-vitamin complex including riboflavin (vitamin B_2_), folate (vitamin B_9_) and cobalamin (vitamin B_12_). For example, lactic acid bacteria fermentation increases riboflavin contents of cereals and legumes by 2.5–5 mg/kg. *Propionibacterium freudenreichii* synthesize vitamin B_12_ in soy or cereal at 0.95–3.2 μg/100 grams. *Bacillus subtilis* natto enhances vitamin k_2_ in fermented soybean products, with reaching higher concentration (< 130–500 μg/100 g) (2, 16, 17). Some of these microbial vitamin productions contribute to the reduction of micronutrient deficiencies, especially in plant-based diets where vitamins are generally very low. Another important pathway in which probiotics increase nourishment is by increasing mineral bioavailability. During the process of fermentation, they form organic acids such as lactic and acetic acid, which lower the pH value of the food. This acidification results in the solubilisation of minerals like calcium, iron and zinc (18). Many strains also release an enzyme called phytase that degrades phytates, the substances in plants that bind minerals thereby reducing their absorption. The result is a significant reduction in phytate during the fermentation. Mineral availability is increased 10%−20% for calcium and 1.5–2.2 times higher when compared with the unfermented foods for iron and zinc. Fermented legumes and vegetables always demonstrate these enhancements, which means that probiotic–fermented foods are effective dietary sources that can be used to mitigate deficiencies in different populations (19, 20).

Probiotics also enhance the quality of the proteins by proteolysis. The lysosomes release proteases which cleave complex proteins into smaller peptides and free amino acids. This process increases essential amino acids such as lysine and leucine which are normally the deficient nutrient in cereals and legumes. In fermented chickpea flour lysine content increases from approximately 28.7 to 110.9 mg/kg; the nutritional value and digestibility of the protein is markedly increased. In the case of lentils the protein digestibility is also increased during the fermentation which affects from about 21.33 to 25.02%, which supports better nitrogen retention and utilization of nitrogen in the consumer (21, 22).

**Figure 3:**
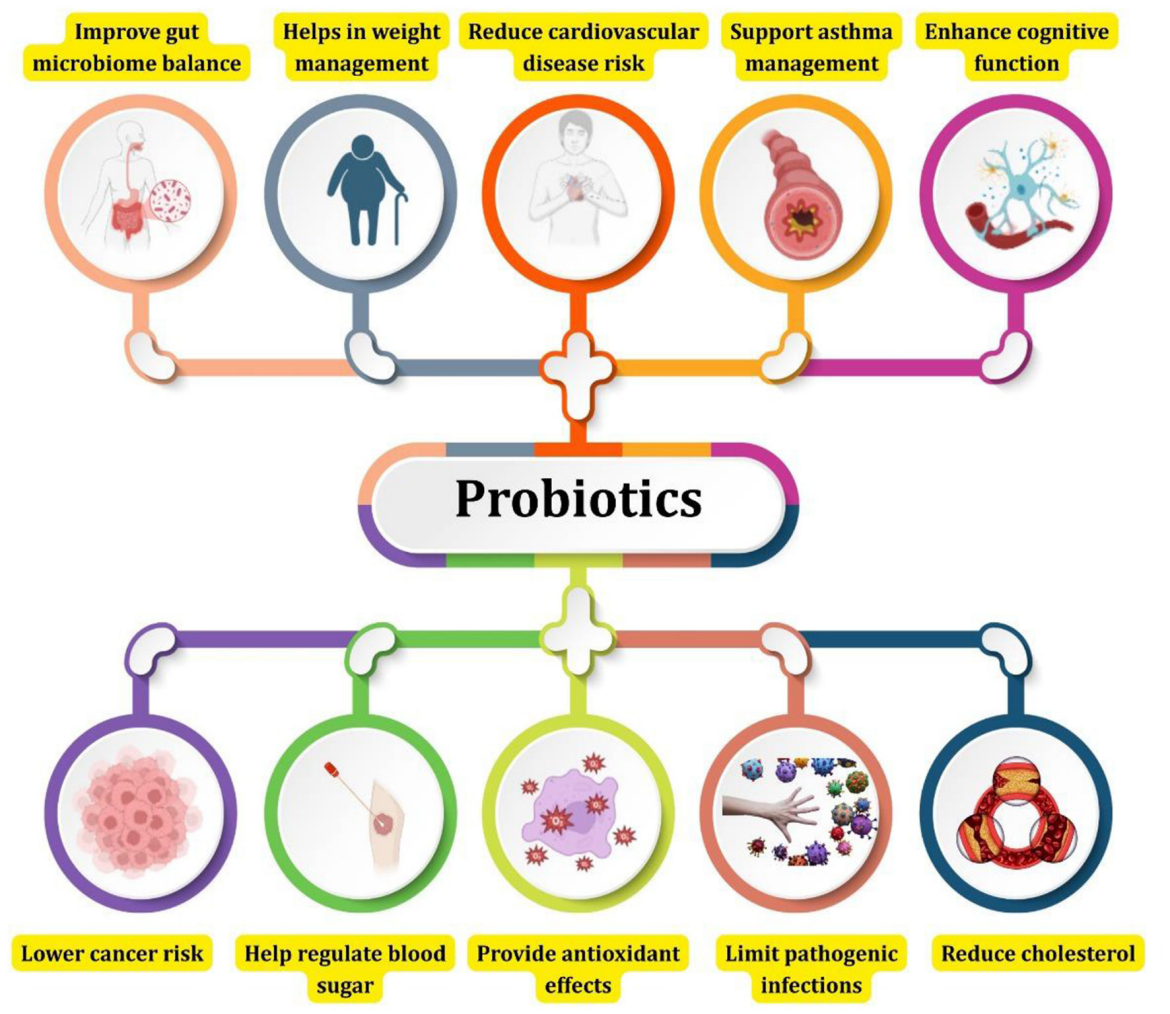
Role of probiotics in human health.

Functional metabolites are also produced during fermentation [gamma-aminobutyric acid (GABA), exopolysaccharides and short-chain fatty acids (SCFAs)]. GABA has neuroprotective benefits and blood pressure lowering benefits. SCFAs like acetate, propionate, and the butyrate, which are produced by the fermentation of the fibers that are prebiotic, are beneficial to the gut health and to the metabolism in general. The production of these compounds is dependent on the fermentation variables of pH, salt level and substrate. SCFA is at its peak when the dietary fiber content lies in the range of 2%−3% and pH lies in the range of 4.2 and 2% NaCl for maximum of GABA (23). In addition to synthesis of specific nutrients, probiotics improve nutrient absorption through the effects of probiotics on gut physiology and immunity. They exclude harmful pathogens, stimulate mucin production to strengthen the intestinal barrier and stimulate anti-inflammation cytokines. These effects help improve the environment of the gut for maximum absorption as a whole. Probiotic colonisationalso supports a balanced colonization of the microbiota, allows the metabolism of complex dietary compounds into absorbable forms and also boosts overall nutritional status. Quality and standardization of probiotic enhanced fermented foods is dependent on careful control of microbial strains, fermentation parameters and substrate composition in order to ensure the consistency of the nutrient enhancement. Standard values documented for nutrient enhancement include riboflavin levels ranging up to 2.5–5 mg/kg vitamin ruthenite irradiation, vitamin B_12_ concentrations ranging from 0.95 to 3.2 μg/100 g, vitamin K_2_ concentrations up to 100 μg/100 g, folate concentrations increased between 150 and 340 μg/100 g, calcium bioavailability increased by 10%−20%, and iron and zinc absorption increased from 1.5 to 2.2 times. Amino acid enrichment is also significant taking levels of lysine more than threefold higher in some fermented legumes. These values are benchmarks for researchers and industry stakeholders looking to develop functional foods with improved nutrient values (24, 25). Figure 3 depicts overall role of probiotics.

## 3 Mechanisms of nutrient enhancement by probiotic microorganisms

### 3.1 Vitamin biosynthesis

Microorganisms produce some vital vitamins such as folate (vitamin B_9_), riboflavin (vitamin B_2_), vitamin B_12_ (cobalamin), vitamin K (menaquinones and phylloquinone). These vitamins are used to power cell metabolism as well as electron transport and many enzymes (Table 1). Production capacity varies remarkably from one breed to another due to sets of genes, regulating genes and environmental interaction. These differences influence microbial communities and facilitate applications in biotechnology, and in nutritional use. The areas in the rest of the technical review describe the biosynthetic pathway, important enzyme reactions, typical kinetic values, and other effects of strain genetics on production of each of the vitamins discussed (26).

**Table 1:** Vitamin enrichment by probiotic fermentation and physiological effects.

**Vitamin**	**Concentration increase (typical range)**	**Food sources**	**Probiotic strains**	**Effect on body**	**References**
Folate (B_9_)	150–340 μg/100 g	Fermented cereals, legumes, dairy, tempeh	*Lactobacillus plantarum, Bifidobacterium longum*	Supports DNA synthesis/repair, fetal development, brain function, immune cell growth	(27, 28)
Riboflavin (B_2_)	2.5–5 mg/kg	Fermented cereals, yogurt, kefir, vegetables	*Lactobacillus plantarum, Streptococcus thermophilus*	Enhances mitochondrial energy generation and antioxidant capacity	(29, 30)
Vitamin B_12_ (cobalamin)	0.95–3.2 μg/100 g	Fermented soy, cereals, miso, cheeses	*Propionibacterium freudenreichii*	Essential for blood cell production, nerve function, prevents anemia	(31, 33)
Vitamin K_2_ (menaquinones)	50–100 μg/100 g (up to 130–500 μg by *Bacillus subtilis* natto)	Natto, kimchi, sauerkraut, cheeses	*Bacillus subtilis* natto, *Lactobacillus plantarum*	Regulates blood clotting, bone metabolism, reduces arterial calcification	(34, 35)

Folate (vitamin B_9_) synthesis is present in bacteria and plants but absent in animals, so it is important to the host nutrition. The folate molecule consists of a pteridine ring, p-aminobenzoic acid (PABA) and glutamate groups. The pathway is initiated by Chorismite synthesized via the shikimate pathway derived in turn from erythrose-4-phosphate and phosphoenolpyruvate of the pentose-phosphate and the glycolytic pathways. An example of a set of mostly adjacent genes are pabA, pabB, pabC (for the assembly of PABA) and folE, folB, folK, folP, folC, folA (for the assembly of pterin and folate). From chorismite to PABA, then to dihydropteroate, and then tetrahydrofolate by dihydrofolate reductase (DHFR) in the *de novo* synthesis of this compound, which needs NADPH. DHFR has a Km of about 1–10 μM and the turnover number (k_cat_) of 1–10/s representing efficient catalysis (2, 27, 28). Strain specific variation is extreme. Complete folate operons are found in certain types of lactic acid bacteria, and significant amounts of 10–100 μg/g in folate are found in fermented foods, while others lack key genes and are folate auxotroph's. The expression is further controlled by horizontal gene transfer and regulatory networks which influence the yield of folate as well as the adaption to the folate content of the environment. These variations allow symbiotic relationships, with folate producing strains providing for the health of gut and host nutrition (28).

Riboflavin biosynthesis is conserved in bacteria, fungi and plants, where subtle distinctions exist in the manner in which the pathway is fluently and each is regulated. The reactants begin with GTP and ribulose chain phosphate (ribulose-5-phosphate) which is part of the pentose-phosphate pathway. The genes for the enzymes mediating the major steps are contained in the rib operon (known commonly as ribA, ribB, ribD, ribE, and ribH), the key enzyme intermediates are GTP cyclohydrolase II in which GTP is converted to 2,5-diaminoribosylamino-4(3H)-pyrimidinone5-phosphate and finally riboflavin is produced through riboflavinato-2,5-dioxo-4-aminopyrimidine and 5-amino-6-(D-ribitylamino)uracil (27, 29). Activity data for riboflavin synthase shows that it has a kcat of 0.5–5/s and Km from 10 to 50 μM, which represents an appropriate balance between catalytic efficiency and substrate affinity. The level of production varies from microbes, engineered *Bacillus subtilis* strains can reach more than 20,000 mg/L during fermentation whereas values usually remain below 1,000 mg/L in the wild type. Metabolic engineering of E. coli taken from the purine and pentose phosphate pathway (PPP) pathway can provide redirection to riboflavin precursor flux. The differences between strains are due to differences in promoter strength, gene copy number, feedback inhibition and in gene capacity to recycle cofactors such as NADH. These factors have been exploited to improve the industrial yield of vitamin B_2_ and to increase its dietary supply (29, 30).

Vitamin B_12_ production is incredibly complex and it is limited to only certain bacteria and archaea. The pathway involves many enzymatic steps in the synthesis of a corrinoid ring using cobalt. Aerobic and anaerobic diverge in two major ways, in how and when cobalt is inserted. Starting from aminolaevulinic acid formed from glutamate, the pathway goes through the uroporphyrinogen III and precursor precorrin molecules before the aerobic cobaltochelatase enzymes introduce cobalt. Cob A is responsible for cobalt absorption as well as following enzymes such as CobI, CobM, CobF, and CobN (31, 32). *In vitro* activity of these enzymes is in range of 0.1–5/s associated with low micromolar K_m_ values suggesting high specificity for substrates. Production suffers from great variations industrial *Propionibacterium freudenreichii* strains are capable of 10 mg/L, whereas other strains don't produce almost anything. Genetic differences include presence or absence of complete operons, oxygen and cobalt responsive regulation and cobalt transport systems. Synthetic biology has succeeded in transferring entire B_12_ gene clusters into non critiques for which they produce. *De novo* synthesis in once incapable strains (33).

Vitamin K synthesis in microorganisms, particularly with respect to menaquinones (vitamin K_2_), goes through the menaquinone biosynthesis (MK) pathway that originates from the shikimate pathway to isochorismate. The number of isoprenoid units determines the length of the side chain of vitamin K_2_ (e.g., MK-4, MK-7). Enzymes such as MenF, MenD, MenH, MenC, MenE, MenB, and MenA cause the sequential uptake of isochorismate to DHNA (1,4-dihydroxy-2-naphthoate) and menaquinone (28, 34). The kinetic data for MenA, the prenyltransferase of DHNA, are K_m_ values (around 10 μM) and adequate kinetics rates for efficient conversion. Strain variations are large engineered strains *Bacillus subtilis* have been shown to produce more than 40 mg/L MK-7 in fermentation experiments (plastic strains reproduce lower levels of MK-7 than natural strains). Variability is taking place due to variation in genetic polymorphisms affecting enzyme expression and stability. The variety of menaquinones and strain specificity affects the electron transport of bacteria and of the host status of vitamin K. Some commensals in the intestinal tract are important contributors of vitamin K (35, 36).

While different types of vitamin biosynthesis pathway have the same basic biochemical and genetic features, they differ greatly at the strain level because of the evolutionary pressure, gene cluster structure, regulatory network, and the adaptation to the environment. The flux through these pathways is carefully regulated through feedback inhibition, transcription, fluxes or substrate availability with cofactors like the NADP, ATP and metal ions necessary for the enzymatic reactions. Strain-specific characteristics are of paramount importance to biotechnology strain selection or engineering to overexpress pathway genes and eliminate competing branches makes for increased yield. The metabolic circuitry of these metabolic pathways is integrated with the central metabolic networks i.e., glycolysis, pentose phosphate pathway and the TCA cycle, to keep a check between growth and synthesis of vitamins. Industrially, efficient producer strains routinely produce somewhere in the range of from 10 to 1,000/L as proof of principle for a real food supplement or pharmaceutical agent based on microorganism-produced vitamins. Continued study of the fields of enzymology, strain genetics and metabolic engineering expands the scope of our ability to use these pathways for public health and industrial applications (29, 35, 37).

### 3.2 Mineral bioavailability

#### 3.2.1 Phytate degradation and organic acid production

Fermented foods enhance the absorption of minerals due to the breakdown of phytate by the probiotics and the production of organic acids. These two actions work together to counteract the antinutrients that typically are the limiting factor in mineral uptake in the digestive tract. Phytate (myo-inositol hexakisphosphate) is present in cereals, legumes, nuts and seeds. It strongly binds minerals such as calcium, iron, zinc and magnesium forming insoluble complexes thus reducing their availability that are beneficial for health (Table 2). This antinutrient effect interferes with mineral absorption and causes widespread micronutrient deficiencies, particularly in individuals who consume mostly plant foods. Probiotics with the addition of phytase enzymes, break down phytate into smaller inositol phosphates, free phosphate and finally myo-inositol. This process is responsible for releasing minerals that were bound to phytate, making them more soluble and therefore, easier to absorb (38).

**Table 2:** Mineral bioavailability improvements and health impacts.

**Mineral**	**Improvement mechanism**	**Absorption increase**	**Food matrices**	**Effect on body**	**References**
Calcium (Ca^2+^)	Phytate degradation + organic acid solubilization + peptide chelation	10%−20% increase	Fermented dairy, legumes, cereals, nut milks	Supports bone density, metabolic function, muscle contraction	(38, 39, 41)
Iron (Fe^2+^)	Phytate breakdown, organic acid reduction of Fe^3+^ to Fe^2+^, siderophores	1.5–2.2 times increase	Fermented cereals, vegetables, legumes	Improves oxygen transport, prevents anemia, supports immune function	(17, 41)
Zinc (Zn^2+^)	Phytate hydrolysis + chelation by organic acids	1.5–2.2 times increase	Cereals, legumes, vegetables, nut milks	Enhances immune system, growth, wound healing, enzyme function	(41, 42)
Phytate (antinutrient)	Breakdown by microbial phytases, acid-induced mineral release	Up to 70%−80% reduction	Cereals, legumes, nut milks, vegetables	Removal of mineral absorption inhibitors, improves overall mineral uptake	(47, 48)

Probiotic phytases of species, particularly *Lactobacillus plantarum, L. brevis, Bacillus subtilis* natto and *B. coagulans* are highly specific for sodium phytate while efficiently degrade it in fermented foods. Their molecular weights are 38–73 kDa. Ca^2+^ ions are required for the binding and action of these on the substrate. Different strains have different optimum pH and temperature, but all of them use ionic and hydrogen bonding in their active sites and have multiple Ca^2+^ ions stabilizing the enzyme. During fermentation these enzymes can eliminate up to 60%−80% of the phytate, and this greatly reduces the mineral binding effect. At the same time, the fermentation process also produces the addition of organic acids, principally lactic, acetic, propionic and butyric acid by metabolizing carbohydrate. The catalytic mechanism in phytases is based on ionic and hydrogen bonds in the active site, which is stabilized by a number of Ca^2+^ ions for fine tuning the substrate specificity. These acids reduce the pH of the food matrix, which increases the solubility of minerals by increasing anion solubility of those minerals due to protonation and disruption of phytate-mineral complexes (39). The acidified environment further stimulates both the host and microbial phytases with further enhancement of mineral liberation. As a result, the increased acid levels result in increased bio accessibility to minerals; infact calcium bioavailability may increase by 10%−20%, and iron and zinc absorption by 1.5–2.2 times compared to unfermented foods. Short-chain fatty acids also maintain intestinal health and increase intestinal mineral absorption (40).

Studies show that there are probiotic strains with high phytase activity to increase mineral content in animals and probably in humans. For example, *Lactococcus lactis* psm16 was more effective at the hydrolysis of phytates to increase levels of minerals in mouse tissues indicating a better overall bioavailability. These strains offer potential at combating deficiencies of the mineral type. The balance of phytate breakdown and production of organic acids depends on conditions of fermentation type, substrate, pH, temperature, and viability of microorganisms. Optimizing these factors is critical to getting minerals to be as well as fortifying as possible (41, 42). In real world trials too, the bioavailability of minerals references such as calcium increment rates often increase by 10%−20% and iron and zinc increase often by 1.5 to more than 2 times compared to that of the unfermented food. The amount of phytase activity varies between strains but they all exhibit fast and stable hydrolysis which remains active within the gastrointestinal tract and ensures that minerals are released within the body. Thus, the simultaneous action of phytate degradation and organic acid production provides a natural and sustainable approach for increasing the state of micronutrients, which is most important for plant-based diets prone to developing deficiencies in micronutrients (43).

In the microbial nutrient enhancement of fermented foods, short chain fatty acids (SCFAs) including acetate, propionate, and butyrate fats are produced by the action of probiotics in fermenting complex carbohydrates such as dietary fibers and resistant starches. These processes of making mimic the conditions in the large intestine, where the non-digestible sugars are broken down by lactic acid bacteria (*Lactobacillus, Bifidium*) and butyrate producing microbes (*Roseburia, Faecalibacterium*) into pyruvate. From pyruvate they funnel carbon into acetate by phosphotransacetylase and acetate pyruvate kinase, propionate by succinate and propionate pathways or succinate by propionate pathways, and butyrate by butyrate on butyl-CoA acetate, CoA butyrate transferase or by crotonate on butyrate pathway which involves cross feeding of microbial community (44, 45). Animal studies of foods enriched with probiotics (e.g., pearl millet or soy inoculated with probiotic strains) reveal an increase in SCFAs in the cecum: co-culture of *Bifidobacterium bifidum, Bifidobacterium longum* and *Lactobacillus rhamnosus* increase total SCFAs by 1.5–2 times, compared to controls, and butyrate by 35%, via an increase in the abundance of Clostridial. This is correlated with increased thickness of mucus layer and expression of mucin-2 due to the inhibition of HDAC. Human studies of fermented dairy or plant food sources reveal increases of 20%−29% fecal butyrate after 4 weeks and associations with higher Ruminococcusbromii activity and degradation of fiber which reduces levels of inflammation markers such as IL-6 by the 15%. Foods fortified with iron or zinc also increase SCFA production by modifying the microbiome, enhancing the absorption of nutrients and also enhancing gut barrier functions in models of deficiency. These mechanisms point to the therapeutic potential of nutrient- enhanced ferments (44, 46).

### 3.3 Chelation and improved absorption of iron, zinc, and calcium

Chelation and increased absorption of important minerals such as iron, zinc and calcium is significantly increased in fermented foods. Probiotic microorganisms produced during fermentation mediate such benefits. These minerals are vital to numerous physiological functions of the body, this includes oxygen delivery, immune help, chemical courts, and bone health but often are not well-available in plant-based diets due to their binding to antinutrients such as phytates and polyphenols. Fermentation overcomes these barriers by producing organic acids and certain chelating agents which dissolve the minerals and break up the inhibitory complexes and facilitate intestinal absorption, thereby helping correct common micronutrient deficiencies all over the world. Chelation occurs when microbial metabolites bind to the mineral ions and keep them in a soluble bioavailable state and prevent precipitation and complexing with inhibitors in the diet. Probiotic strains such as various types of lactobacilli and bifidobacteria produce organic acids such as lactic acid, acetic acid, and other substances which act as natural chelating agents. By reducing the pH and forming mineral-organicacid complexes, the solubility of iron (Fe^3+^/Fe^2+^) is increased, as well as the solubility of zinc (Zn^2+^) and calcium (Ca^2+^) and the release of these cations from insoluble phytate or tannin complexes of which cereals and legumes are abundant. The pH drop between 3.5 and 4.5 during fermentation increases solubility and absorption of calcium (41, 42).

Probiotic activity also leads to enzymatic degradation of phytates by phytase enzymes to free up minerals that were previously bound in insoluble forms. The combined effect of phytate hydrolysis and organic acid mediated chelation significantly increases the bio-accessibility of minerals. Studies report up to 2.2 times increase in the absorption of zinc and approximately 1.8 times increase in the absorption of iron have been reported in fermented vegetables as compared with raw versions. Calcium bioavailability has been shown to be greater by 10%−20% in fermented legumes that correspond with low phytate and high acidity. The results of these quantitative improvements are validated through in bond investigate models, animal feeding tests, and human observational investigations, indicating that fermentation is nutritionally important (38, 39, 41). The benefit of probiotic in iron absorption is twofold. First, organic acids help in reducing ferric iron (Fe^3+^) to the more absorbable ferrous state (Fe^2+^), which is preferentially transported across the intestinal epithelium. Second, some probiotic strains produce siderophores and iron-binding peptides, which have the further advantage of solubilizing and facilitating the transport of iron. The synergies between the breakdown of phytate and the microbial metabolites have produced a more favorable environment in the gut for iron uptake and have been associated with increased hemoglobin and ferritin in people whose diets regularly include fermented staple foods (17, 41). Often bound in insoluble zinc phytate, zinc is enhanced significantly by microbial phytate degradation and acid chelation. The resultant rise in free concentration of zinc ions enhances intestinal absorption through zinc specific transporters. Experimental data has been consistently shown that probiotic treated cereals and legumes have 1.5 to over 2 times higher zinc bioavailability than controls (unfermented), which has direct benefits in terms of immune function and growth in vulnerable groups. Calcium absorption is facilitated by the production of acid and hydrolysis of phytate. Organic acids dissolve calcium phosphate complexes and displace calcium from phytate binding sites and probiotics may also up-regulate calcium binding proteins of the gut lining, facilitating uptake. Fermentation of soy, millet, and chickpeas using *Lactobacillus plantarum* has increased calcium solubility and absorption by about 10%−20%, respectively that can help maintain bone health and metabolic functions (47, 48). Peptide—mineral chelation in probiotic, fermented food are affected by microbial proteases (e.g., PepO/X from lactic acid bacterium) which cleave proteins into short peptides (2–6 amino acids, 500–2,000 Da). These peptides are chelated by bonds formed between carboxyl groups, amino groups, and side chains of constituents (i.e., glutamate, aspartate, phosphoserine) and Fe^2+^, Zn^2+^, or Ca^2+^. This interaction leads to increased mineral solubility and absorption 2–3 fold more than is through DMT1 and PEPT1 co-transport in the gut and sensitivities to inhibitors such as phytate while facilitating transported minerals and not dispersed (49).

### 3.4 Amino acid and protein enrichment

Proteolytic activity during probiotic fermentation plays an important role for enhancing the nutritional and functional quality of fermented foods through enrichment in amino acids and proteins. In plant and dairy products, proteins are often complex molecules, extremely large and not easily digested and will not give you enough of essential amino acids. Probiotics, in particular lactic acid bacteria (*Lactobacillus plantarum* and *Lactobacillus helveticus*), and certain species of Bacillus have available a variety of proteolytic systems. These include extracellular proteases, cell wall-bound proteinases, and intracellular peptidases which act in series and concert with one another to degrade complex proteins into smaller peptides and free amino acids (50). The result is increased digestibility of protein, and more amino acids because for instance, the extracellular proteases break down whole proteins into oligopeptides. Those are then sliced into short peptides and free amino acids by cell envelope proteinases and intracellular peptidases. How well such a cascade works depends upon the strain, as well as environmental conditions such as pH, the substrate and the way the fermentation is set up. The result of the proteolysis is an output of not only amino acids, but also bioactive peptides small amino acid chains that have a lot of positive health benefits (Table 3). These peptides have been shown to reduce blood pressure, combat oxidation, modulate immunity, kill microbes, and act like opioids. For example, peptides produced from fermentation with *L. helveticus* have a great inhibitor of angiotensin converting enzyme, assistance in the control of high blood pressure. Plant protein derived bioactive peptides also have the ability to scavenge free radicals, bind metal ions and modulate immune responses. The types of peptides and their impact on the probiotic depend on the profile of the protease present in the probiotic and the source of protein (51, 52).

**Table 3:** Amino acid and protein enhancement by fermentation and functional benefits.

**Parameter**	**Increase**	**Food sources**	**Probiotic strains**	**Effect on body**	**References**
Essential amino acids (lysine, leucine)	2–4 times increase (e.g., lysine from ~28.7 to ~110.9 mg/kg in chickpeas)	Legumes, dairy, cereals	*Lactobacillus plantarum, L. brevis, Bacillus coagulans*	Supports muscle development, tissue repair, neurotransmitter synthesis	(53, 60)
Protein digestibility	10%−15% increase	Lentils, legumes, cereals	Lactic acid bacteria including *L. plantarum, L. helveticus*	Improves nitrogen utilization, reduces protein malnutrition	(50)
Bioactive peptides	50–300 mg/100 g	Dairy, fermented foods	*Lactobacillus helveticus, Streptococcus thermophilus*	Antihypertensive, antioxidant, antimicrobial, immunomodulatory	(51, 52)

Different probiotics strains express different gene clusters of proteases, such as aminopeptidases, endopeptidases and di/tri-peptidases. Genes for cell-envelope proteinases are widely found among *L. helveticus*, which makes casein breakdown very efficient, whereas other species exhibit more variation. Because of this genetic diversity, proper selection of strain used and control of fermentation is necessary holistically maximize the output of amino acids and bioactive peptides. More proteolysis can be promoted by mixing complementary strains. After the foods are fermented, there are often more free amino acids in the foods (53, 54). For instance, chickpea flour fermented with *L. plantarum* and *L. brevis* increases lysine level from 28.7 to 110.9 mg/kg and leucine (a branch of lysine) from 38.9 to 129.8 mg/kg that complete deficiencies seen in cereal and legume diets. Lentil protein's digestibility is also increased fromapproximately 21%−25% giving improved use and better effectiveness in nitrogen. These protein increases are also accompanied by improved flavor, texture, and storage duration that developers could eat, due to the presence of antimicrobial peptides, which make the products more desirable to the consumer (55).

Proteolytic action also enhances the availability of the minerals. Amino acids and peptides that are released during fermentation can be used for binding to minerals such as calcium, magnesium and zinc to create soluble complexes, which are easier for the body to absorb. Casein phosphopeptides are produced by the proteases of probiotic organisms have been shown to be particularly effective in fixing calcium, to bone health. In total, it is possible to increase the level of essential amino acids by 2–4 times during the fermentation process. Digestion improvement for protein in legumes and cereals are in most cases 10%−15% or more. Concentrations of bioactive peptides may vary between 50 and 300 mg/100 gm of saponin, necessitating the process employed (56, 57). Probiotics also produce essential amino acids like lysine, leucine, isoleucine, valine, methionine, threonine, phenylalanine, tryptophan and histidine that human beings cannot manufacture on their own. These amino acids are often deficient in plant based foods, and they are synthesized by the cells from central carbon metabolites such as pyruvate and alpha-ketoglutarate. Enzymes in the form of acetolactate synthase in *Bacillus subtilis* and Lactobacillus help synthesize branched chain amino acids, especially in the event of nutrient restrictions (57–59).

Lysine is synthesized primarily by the diaminopimelate pathway which is well-known in *L. plantarum* and *Bifidobacterium bifidum*. When legumes are cured with such microbes, lysine can be increased almost four times, correcting cereal-based diets. Sulfur rich amino acids such as threonine and methionine also increase during probiotic fermentation. Tryptophan is produced through the routine of different shikimate and an anthranilate and degrees depend on the complete manufacturer. Engineered or robust strains of *L. reuteri* are able to increase levels of tryptophan during the fermentation process, which helps neurotransmitter and protein synthesis. Studies confirm big increases in both inclusion both free and total essential amino acids in soy, cereals and other foods. Leucine can get to 130 mg/kg or so with other essential amino acids rising significantly as well (53, 60). These improvements are due to both the new synthesis and the proteolytic release and are always consistent at many fermented foods (legumes, cereals, dairy food, etc.) using strains such as *L. plantarum, L. casei*, and *Bacillus coagulans*. The way amino acid production is regulated is strain specific, depending on the presence of the genes, the amount of enzymes produced, and also external factors like the type of food components that can be found in the environment (61). Some strains do not contain complete pathways, but culturing co-operational bacteria may supply a balanced amount of amino acids. Further increases in essential amino acid synthesis by adding nutrients or precursors to the fermentation medium are possible. From a health standpoint, the addition of these amino acids to fermented foods helps with muscle development, tissue repair, immunity as well as neurotransmitter balance, which particularly concerns the vegetarian and vegan population, where a deficiency is likely. Aminoacidrich fermented products can also be used to control sarcopenia, malnutrition, and chronic diseases through enhancement of general nutrition (62).

## 4 Probiotic strains involved in biofortification

**Figure 4:**
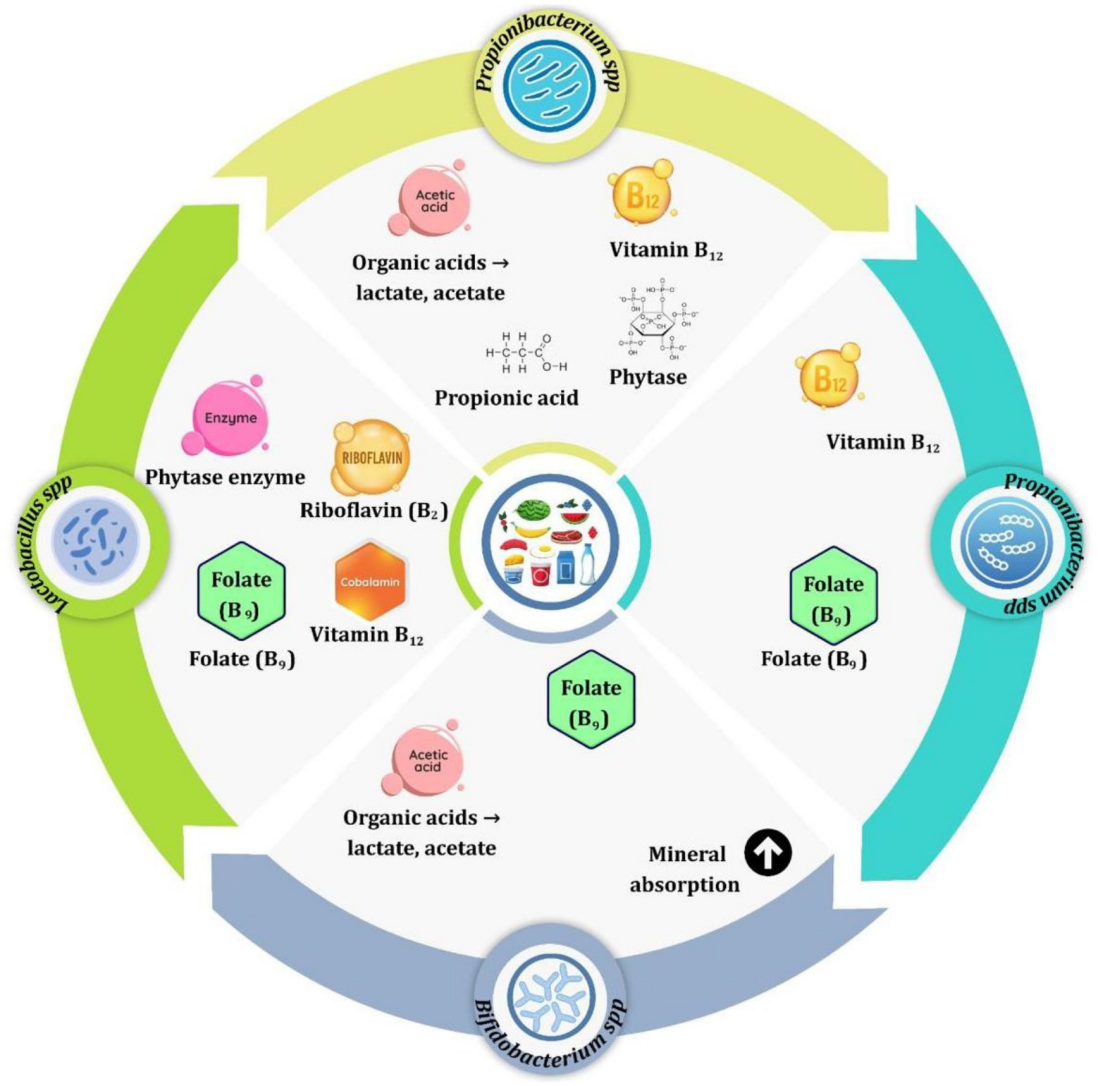
Different probiotic strains that involved in biofortification and its role.

Probiotic strains, like bifidobacteria and other members of *Lactobacillus, Bifidobacterium, Propionibacterium*, and *Streptococcus thermophilus* are involved and indispensable for biofortification of fermented foods. They enhance these foods by creating vitamins, producing enzymes, and enhancing nutrient uptake. The outcome is increased vitamin content, better digestion, and increased absorption of minerals and proteins, which creates functional foods addressing micronutrient deficiencies. Lactobacillus types are the most versatile and best-used probiotics in this regard. The amounts they produce depend on the strain as well as the raw material (2, 17). In addition to vitamins many strains of Lactobacillus produce phytase, an enzyme that digests phytic acid that is a common antinutrient and in turn increases the availability of calcium, iron, and zinc. Their intensive proteolytic activity also contributes to the further improvement of protein quality, and essential amino acids such as lysine, leucine are liberated during fermentation. Because these functions are strain specific, manufacturer can develop a customized biofortification strategies for specific nutrients and food matrices (63, 64) (Figure 4).

Bifidobacterium species have a complementary relationship with Lactobacillus, particularly in the production of folate and the gut health. Bifidobacterium longum subspecies longum strain and Bifidobacterium breve have high folate enhancement rates for dairy and plant based fermented foods, giving 150–320 μg/100 gm; similar to Lactobacilli. They also produce organic acids, which cause a decreased pH in the food matrix, which lessens the solubility of minerals and degrades phytate levels. Although Bifidobacteria have less pronounced proteolytic activity than Lactobacillus, they favorably influence the bioavailability of nutrients indirectly, as they influence gut microbiota, leading to the increase in the absorption of nutrients produced in the gut. These synergistic roles highlight the key role of bifidobacterium in functional food biofortification (65, 66).

Propionibacterium species specifically *Propionibacterium freudenrichii* have become well-known as a source of vitamin B_12_, one of the vitamins seldom created by other probiotics. This ability is important in the case of plant-based foods that lack natural sources of B_12_. The strain can enrich soy beverages and cereals with 0.95–3.2 μg/100 gm which provides amounts of vitamin B_12_ equivalent to recommended dietary allowances. Propionibacterium also produces propionic acid which further acidifies the food matrix and stimulates mineral availability. While their proteolytic and phytase activities are limited and lower than that of Lakobacillus, the importance of Propionibacterium in providing B_12_ as well as enhancing mineral availability is of strategic significance in vegetarian and vegan populations (10, 67, 68). *Streptococcus thermophilus* is used extensively in the fermentation of yogurt and cheese. Although the level of vitamin production is very low in comparison to Lactobacillus and Bifidobacterium, it has a part to play in biofortification if used in mixed cultures with *Lactobacillus delbrueckii* subsp. Bulgaricus (68). This coaction results in greater production of folate and riboflavin. The acid produced by *S. thermophilus* decreases the pH, increasing solubility of minerals for absorption. *S. thermophilus* also has a moderate proteolytic activity and assets in the release of amino acids and bioactive peptides as a complement in the enhancement of nutrients. Its fast fermentation kinetics and adaptability to the substrate make it a useful starter culture for the improvement of the nutritional quality of dairy products (69, 70). Safety assessment frameworks used for engineered strains for microbial biofortification of fermented foods concentrate on risk-based assessments for the protection of human, animal and environmental health. These frameworks are based on QPS of EFSA and FAO/WHO guidelines. QPS applies to well-characterize taxa like Lactobacillus and bifidobacterium used for enrichment of foods with riboflavin or vitamin B_12_. To qualify strains must have clear taxonomy, a safe historical record, no virulence of antimicrobial-resistance genes and prove acceptable for food use. Strains that do not conform with QPS criteria are whole genome sequenced for pathogenicity, toxin production, and assessment of the potential for horizontal gene transfer (71, 72).

Comparative studies have shown that those genera have unique and complimentary nutrient-enhancing capacities. Lactobacillus strains such as *L. plantarum* and *L. reuteri* are very versatile in their performances, secreting in large quantities B vitamins, phytase for mineral release and proteases that enhance the digestibility of proteins, with an increase of 10%−15%. Bifidobacterium is efficient in folate production and uses organic acids to mobilize minerals with its effects on gut microbial carrying over to improved absorption. *Propionibacterium freudenreichii* is specialized in vitamin B_12_ synthesis assisted by propionic acid (68). Platelet-Rich Fibrin is an important plant-based biofortification microbe with a low proteolytic activity. *Streptococcus thermophilus* moderately synthesizes vitamins but is communal in the fermentation process so the production of nutrients is increased by synergistic activities. These genetic and metabolic characteristics differ for different strains. The genetic bases of these biofortification traits are well-characterized (73). Lactobacillus types have gene clusters for the synthesis of B group vitamins—e.g., folP and folA for folate and the rib operon for riboflavin. Many also have some enzymes such as phytase and protease to break down the antinutrients and proteins. Bifidobacterium strains possess a full folate pathway with the production of organic acids which modify the fermentation environment. Propionibacterium encode extensive clusters for cobalamin biosynthesis; more than 30 enzymatic steps are needed for active vitamin and for propionic acid production. *Streptococcus thermophilus* contains a smaller set of folate and riboflavin synthetic genes, acidification, and proteolysis. These genes can be associated with the measurable nutritional improvements associated with Lactobacillus; the increases have been measured as 2.5–5 mg/kg in riboflavin and up to 150–340 μg/100 grams in folate (74, 75). Excessive bifidobacterium imitation is creating similar increases in folate. *Propionibacterium freudenreichii* has the capacity to add 0.95–3.2 μg/100 g of vitamin B_12_. All strains enhance calcium absorption by 10%−20% and enhance iron and zinc absorption by means of organic acid. By utilizing or co-culturing certain probiotic strains, manufacturers can use these complementary characteristics in creating functional fermented foods for micronutrient deficiency. This sustainable strategy addresses the need for more consumer-oriented natural foods with health promoting benefits and has potential to implement scalable, culturally acceptable nutritional interventions globally (68, 76). Genetically modified biofortificants e.g., propionibacterium genetically modified to produce propionate are assessed on a case-by-case basis using the frameworks established by the Office of Economic Cooperation and Development (OECD) and the Food and Safety Authority (EFSA). Key assessment elements include phenotypic stability, antibiotic resistance profiling as well as acute and chronic rodent toxicology. Rodent studies require NOAEs of >10^9^ CFU/kg. Allergenicity is anticipated by ensuring sequence homology with known allergen is less than 35% (71).

## 5 Fermented food matrices and their influence

**Figure 5:**
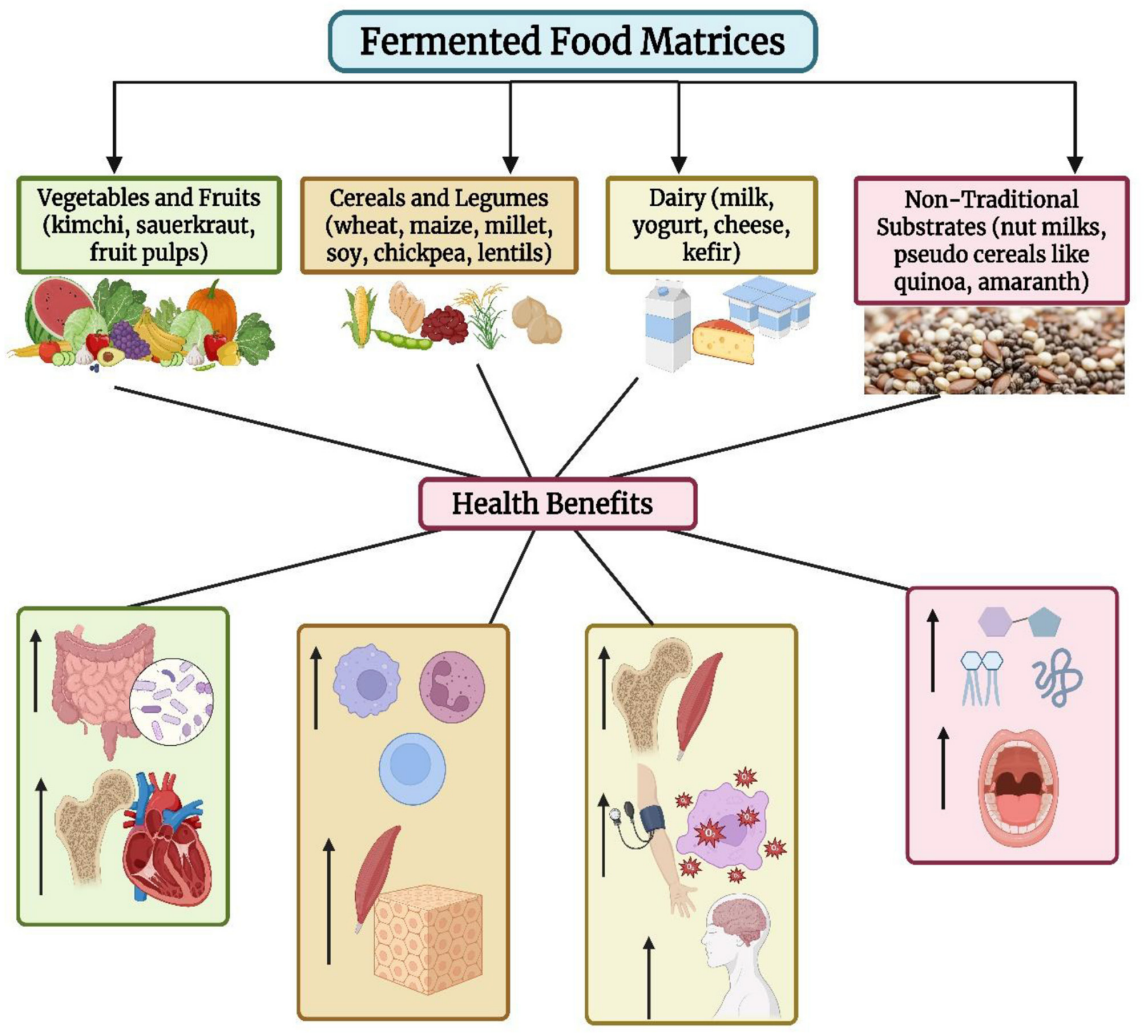
Overall health benefits of fermented food matrices. Created with BioRender.com.

Fermented food matrices strongly influence the efficacy of probiotics to enhance the effects on nutrient and their availability and health benefits. The physical, chemical and compositional characteristics of each of the substrates determine how probiotics grow, metabolize, and synthesize nutrients during the fermentation process. Matrices include dairy, cereals, legumes, vegetables, and fruits each having different macronutrients, antinutrients, pH, water activity, and native microbes. Knowing the role of these characteristics in probiotic biofortification is essential for the adaptation of the nutrient enhancement strategy to various foods and diets (Table 4; Figure 5) (15). Dairy products like milk, yogurt and cheese offer protein-rich, buffered environments providing caseins, whey proteins, carbohydrates, lipids or minerals like calcium. These conditions are conducive to good probiotic growth and metabolic activity, and it is easy for the microbes to produce vitamins (e.g., riboflavin, folate), break down proteins into bioactive peptides and solubilise minerals. For example, the fermentation of milk by *Lactobacillus helveticus* increases bioactive peptide contents by as high as 300 mg/100 g, and increases folate to 200–350 μg/100 g. The high buffering capacity keeps the pH stable and favor microbial enzyme and nutrient synthesis. Calcium bioavailability is increased by 10%−20% in fermented dairy as a result of the action of casein phosphopeptides on calcium binding and intestinal absorption (77, 78).

**Table 4:** Fermented food and their health benefit.

**Food matrix type**	**Typical nutrient changes via fermentation**	**Probiotic metabolic activities**	**Health benefits/effects**	**References**
Dairy (milk, yogurt, cheese, kefir)	– Riboflavin 2.5 – 5 mg/kg – Folate 200 – 350 μg/100 g – Vitamin B_12_ up to 3 μg/100 g in cheese – Calcium bioavailability increased 10%−20% – Protein digestibility increased 10%−15% – Bioactive peptides up to 300 mg/100 g	– Proteolysis of casein releasing essential amino acids and peptides – Phytase and acid production enhancing mineral solubility – Stable pH maintaining nutrient and enzyme activity	– Improved bone health and calcium metabolism – Enhanced muscle repair and immune modulation – Antihypertensive and antioxidant effects from bioactive peptides – Improved vitamin B status supporting energy and neurological function	(82, 83, 86)
Cereals and legumes (wheat, maize, millet, soy, chickpea, lentils)	– Riboflavin 2.5 – 5 mg/kg – Folate 150 – 340 μg/100 g – Phytate reduced up to 80% – Mineral absorption (Fe, Zn, Ca) increased 1.5 – 2.2 times/10%−20% (Ca) – Lysine increased ~3 – 4 fold (e.g., chickpea flour) – Protein digestibility increased 10%−15%	– Phytase degrading anti-nutrients – Organic acid production lowering pH – Proteolytic release of amino acids	– Enhanced iron and zinc absorption aids immune function and growth – Improved protein quality supports muscle and tissue health – Increased B vitamin levels support metabolism and cell function	(87, 89)
Vegetables and Fruits (kimchi, sauerkraut, fruit pulps)	– Vitamin K_2_ 50 – 100 μg/100 g – Riboflavin approx. 2.5 – 5 mg/kg – Folate increases 150 – 250 μg/100 g – Mineral absorption (Fe, Zn) doubled vs. fresh – Enhanced antioxidant metabolites	– Acidic pH favors phytate breakdown and mineral solubilization – Production of organic acids and exopolysaccharides – Biotransformation of polyphenols increasing antioxidant capacity	– Bone and cardiovascular support via vitamin K_2_ – Enhanced mineral uptake improving oxygen transport and growth – Gut health promotion and antioxidant protection – Limited protein enrichment due to low native protein content	(7, 90)
Non-traditional substrates (nut milks, pseudo cereals like quinoa, amaranth)	– Riboflavin 2.5 – 5 mg/kg – Folate 150 – 340 μg/100 g – Phytate reduction up to 70% – Mineral bioavailability increased 10%−20% (Ca) and 1.5 – 2 times (Fe, Zn) – Protein digestibility increased 10%−15%	– Breakdown of complex carbohydrates to SCFAs – Proteolysis releasing free amino acids – Enzyme activity facilitating vitamin synthesis	– Supports gut health and nutrient uptake – Improves micronutrient status especially in plant-based diets – Enhances texture and taste with functional peptides – Addresses protein limitation by combining with complementary sources	(91, 93)

Cereal and legume matrices, which include wheat, maize, millet, soy, chick pea and lentils, are primarily comprised carbohydrates with varying composition of proteins and fiber. Their complex structures can inhibit mineral absorption because their phytate, tannin and polyphenol content. Probiotic fermentation overcomes this by producing organic acids (reduced pH) and enzymes such as phytase, which breaks down 60%−80% of phytates to release minerals such as iron, zinc and calcium (79). Chickpea flour fermentation could increase lysine content from 28.7 to 110.9 mg/kg and increase protein digestibility by 10%−15%. There is also an increase in vitamin levels with riboflavin reaching 2.5–5 mg/kg and folate up to 340 μg/100 g in ferments cereals. The carbohydrate and fiber base works by stimulating the production of short-chain fatty acids which are responsible for supporting gut health as well as nutrient uptake (80, 81). Vegetable and fruit matrices differ as they are more acid (pH around 3–4), have high level of water contained, they are high in phytochemicals like antioxidants and phenolics. These characteristics provide selective environments that affect the survival and activities of probiotics. Fermenting cabbages or cucumbers with *Lactobacillus plantarum* and increases vitamin K_2_ to 50–80 μg/100 g of cabbage and improves phytate breakdown which can double iron and zinc absorption organic acids produced during the fermentation process also contribute to solubilizing minerals and secretion of antioxidant metabolites provide additional benefit to the health. The low protein content that occurs naturally limits enrichment of amino acids compared to dairy and legumes (23, 81).

The microflora native to each matrix further determines biofortification results interacting in competitive or cooperative manners with supplemental probiotics. For example, spontaneous vegetable fermentations contain complex types of bacterial consortia which are able to complement the probiotic enzymes so that the degradation from phytate and the synthesis from vitamins will improve. In contrast, the pasteurized dairy permits control of probiotic dominance for practical nutritional enhancement. Substrate specific prebiotic compounds such as oligosaccharides in legumes favor the growth of bifidobacteria and synthesis of folate, and lactose in dairy products favor Lactobacillus growth and synthesis of riboflavin and subsequently metabolisms (23, 80). Fermentation conditions like the temperature, time, oxygen concentration, and the size of the inoculum interact with traits of the matrix and determine the final product that is biofortified. Optimal temperatures differ based upon matrix and strain but generally lie between 30 and 42 °C for the most efficient production of vitamins and proteolytic activities. Controlled anaerobic or microaerophilic conditions are optimal for phytase expression and organic acid formation which is important for mineral availability. Fermenting for longer periods of time in an alcoholic beer will increase the number of metabolites but may negatively affect sensory properties. Standardization of inoculum size promotes homogeneity in probiotic activity toward every pattern of release of nutrients by a particular matrix (78). Quantitative results are given to demonstrate the effects of matrices, riboflavin reaches 2.5–5 mg/kg in fermented cereals and dairy; folate, 150–340 μg/100 g in a series of legumes and sourdough bread; and at least 3.2 μg/100 g of vitamin B_12_ in fermented soy and 50–100 μg/100 g of vitamin K_2_ in vegetable and legume ferments. Mineral absorption enhancements related to matrix the bioavailability of calcium is increased by 10%−20%, while the bioavailability of iron and zinc are increased by 1.5–2.2 times in fermented cereals and vegetables. Amino acid enrichment is greatest in dairy and legume fermentations resulting in levels of essential amino acids doubling to quadrupling compared with the unfermented base (78, 81).

### 5.1 Dairy-based fermented foods

Dairy originating fermented foods like yogurt, kefir and cheese are the staples in many diets, around the world. They are also good vehicles for microbial biofortification to maintain nutrients while greatly extending their value to human health. These foods are a natural rich source of nutrients in terms of proteins, lipids, vitamins and minerals content and fermentation with probiotic microorganisms mainly lactic acid and yeasts, that increases vitamin content, protein digestibility, mineral bioavailability and produce bioactive compounds. Yogurt is amongst the most widely consumed types of fermented dairy product. It is associated with the strong growth of useful probiotic bacteria such as *Lactobacillus delbrueckii* subspecies bulgaricus and *Streptococcus thermophilus*. Many other yogurts are also supplemented with other species, such as *Lactobacillus acidophilus* and *Bifidobacterium bifidum*, to boost on nutrient profiles. Fermentation generally increases B vitamins such as riboflavin (vitamin B_2_) includes in the 2.5 to 5 mg/kg with the folate (vitamin B_9_) often increasing to 200–350 μg/100 g, and far beyond levels to be found in unfermented milk. Proteindegrading activity enhances digestibility of casein, freeing the essential amino acids, such as lysine and leucine, as well as the bioactive peptides with antihypertensive and immunomodulatory activities. A second major benefit of fermentation is the enhancement of mineral bioavailability; casein phosphopeptides released in the proteolysis enhance calcium bioavailability by approximately 10%−20%. The buffering capacity of the dairy matrix maintains a constant state of pH, which in turn maintains and anchors the nutrients and maximizes the activity of probiotic enzymes (82–84).

Kefir is a complex, probiotic fermented milk product, including symbiotic cultures of bacteria and yeasts, which are *Lactobacillus kefiranofaciens, Lactococcus lactis* and Saccharomyces species. Kefir fermentation does add vitamins; riboflavin concentration is 3–5 mg/kg and folate is 150–300 μg/100 g food. An exopolysaccharide known as kefiran exclusive to the kefir cultures, offers prebiotic effects for supporting gut health. Kefir's complex microbiota produces a more complex proteolytic activity which results in greater release of free amino acids and bioactive peptides—resembling yogurt—enhancing the protein digestibility of around 10%−15%. Mineral absorption is enhanced by organic acids which solubilize calcium, magnesium and zinc. Clinical studies link kefir consumption to the improvement of bone mineral density and immune function as an indicator of improved nutrient bioactivity (85, 86).

Cheese is a variety from fresh soft to aged hard types and is a product which depend on starter cultures of lactic acid bacillus and probiotic adjunct strains to ferment and ripen. During ripening, free amino acids and bioactive peptides are released by proteolytic enzymes and concentrations of lysine and leucine are increased to approximately 120 and 130 mg/kg cheese in aged cheese. Vitamins such as riboflavin rise to up to 4 mg/kg. Certain types of cheese naturally have a high enrichment in vitamin K_2_ (menaquinone) in amounts of up to 50–100 μg/100 g. Vitamin K_2_ is crucial for calcium metabolism. The loss of moisture and buffering effects in the cheese plays a protective role in calcium and phosphorus, which can bring the improvement of calcium absorption of up to 15%. High matrix density and long fermentation time also concentrate bioactive compounds and increase the role of cheese as functional food (83, 84). Dairy fermented food standardized nutrient retention and enhancement values are as follows, the content of riboflavin increases from negligible levels in raw milk to 2.5–5 mg/kg in yogurt and kefir. Folate is increased from 25 μg/100 g of milk to 150–350 μg/100 g after fermentation. Vitamin B_12_ in cheese may reach 1.5–3 μg/100 g, and so this cheese can really contribute to dietary adequacy. Calcium bioavailability is improved 10%−20% as the result of peptides of casein origin. Protein digestibility is increased 10%−15% in yogurt and kefir with corresponding increase in essential amino acids available (82).

### 5.2 Plant-based fermented foods

Plant-based fermented foods including cereals such as dosa and injera, legumes such as tempeh and miso and vegetables such as kimchi and sauerkraut are crucial contributors to world nutrition. They are also vehicles for microbial biofortification based on probiotic fermentation. These foods not only ensure long shelf life but also increase the sensory parameters, having an intrinsic transformation into products with high nutritional value with higher vitamin value, an improved availability of minerals and a better quality of proteins. Probiotics, in the form of mostly lactic acid bacteria and selected yeasts, are responsible for these changes. Fermented cereals such as dosa (made from rice and black gram) injera (traditionally made from teff) undergo a natural or controlled kinds of fermentation that increases the nutrient behavior (16). The predominant microbes are those within the lactic acid bacteria (e.g., *Lactobacillus plantarum, Lactobacillus fermentum*), and produce the B vitamins (such as riboflavin, folate, etc.). A boost in riboflavin levels to 2.5–5 mg/kg and content of folate level to 150–340 μg/100 g of cereals are seen, much more than in raw grains. Fermentation also decreases the phytates (by as much as 80%), which releases calcium, iron and zinc, making absorption between 1.5 and 2.2 times that of uncooked grains. Proteolytic activity results in the release of essential amino acids like lysine and leucine which increase the protein digestibility level by roughly 10%−15% and addresses common amino acid limitations (23, 87).

Human (Carnivore) Nutrition Fermented foods such as tempeh and miso are legume-containing products that use the power of microbial fermentation to enhance the nutrient content. Tempeh is made by *Rhizopus oligosporus* fermenting soybean and this enhances the bioavailability of isoflavones and solubilizes minerals due to the degradation of phytate. Lactobacillus species helps in the synthesis of folate; an increase from a diet of less than 30 μg/100 g compared with raw soy to over 150 μg/100 g when fermented soy was consumed in the form of tempeh. Miso fermentation, with participation of *Aspergillus oryzae* and lactic acid bacteria, provides enrichment of vitamin B_12_ in 1.5–3 μg/100 g in order to meet dietary cobalamin shortfalls. Fermentation also produces bioactive peptides which have antioxidative and antihypertensive activity. Mineral absorption of food in tempeh is improved from 10 to 20% for calcium and iron due to organic acids that reduce pH and increase solubility (16, 87–89).

Vegetable ferments such as kimchi and sauerkraut contain a great deal of vitamins and minerals which are enhanced by the presence of lactic acid bacteria such as *Lactobacillus plantarum* and *Leuconostoc mesenteroides*. Fermentation increases vitamin K (menaquinone) to 50–100 μg/100 g; as well as folate and riboflavin. The low pH (apx.4) favors the breaking down of phytate and solubilization of minerals, resulting in the double absorption of iron and zinc compared with fresh vegetables. Fermentation also serves to produce organic acids, exopolysaccharides and peptides which exert an anti-biotic activity, favoring the gut health and the general metabolism (16, 23). Increasing antioxidant capacity due to this process the bioavailability of polyphenols is increased and the formation of new metabolites occur. Standardized study values confirm these biofortification effects an increase in riboflavin levels of 2.5–5 mg/kg in cereals and vegetables; an increase in folate levels to 150–340 μg/100 g portions in cereals and legumes; an increase in vitamin B_12_ levels of short-to-medium fermentation legumes such as miso of 1.5–3.2 μg/100 g portion; an increase in vitamin K_2_ levels of proven spaces of 50–100 μg (7, 90).

### 5.3 Non-traditional substrates

Non-traditional substrates like fruit pulps, nut milks and novel plant-based matrices are upsetting the paradigm of microbial biofortification by means of fermentation. These substrates provide a myriad of carbohydrates, types of fibers, vitamins and minerals that provide unique habitats for the growth and metabolism of probiotics. They in turn influence the final nutrient profile of the fermented product. Growing consumer interest in plant-based as well as allergen free foods and consumer interest for sustainable foods is driving research to optimize these fermentation processes to maximize biofortification (91). Fruit pulps-mango, papaya, apple, and berry pulps contain a great amount of simple sugars, vitamin C and phytochemicals polyphenols and antioxidants. In the case of fermentation with particular strains such as *Lactobacillus plantarum* and *Lactobacillus rhamnosus*, various shades of these pulps acquire B group vitamins riboflavin to 3–5 mg/kg, folate to 150–250 μg/100 g. Fermentation is also known to increase the antioxidant capacity through microbial biotransformation of polyphenols. The final effects of the production of organic acids is a pH decrease, thus, resulting in better mineral solubility and bioavailability. Complex carbohydrates are broken down into short chain fatty acids that are beneficial to gut health. However, fruit pulps are low in protein hence the amino acid content is low. A more balanced nutrition profile can be achieved by blending with protein rich matrices or supplements with protein (92, 93).

Nut milks, including almond, cashew, and soya milk, are good sources of lipids, moderate protein, vitamin E and unsaturated fatty acids. Probiotic fermentation with species of Lactobacillus and Bifidobacterium increasevitamin B production and free essential amino acids in a process known as proteolysis. The dose of riboflavin may be increased to 2.5–4 mg/kg, with folate increased by 100–200 μg/100 g. Fermentation also reduces the level of phytate by as much as 70% by means of phytase activity, and increases calcium, iron and zinc availability by as much as 20%. The process generates bioactive peptides and polysaccharides that enhance texture, taste and functional benefits, for example, modulation of immune systems (3, 93).

Novel plant-based matrices such as fermented pseudocereals (quinoa and amaranth), legumes (mung bean, chickpeas) and seed-based substrates (chia and flaxseed) are gaining interest because of their high level of protein, fiber and micronutrients. Fermentation with strains such as *L. plantarum* and *Bifidobacterium longum* has resulted in an increase in vitamin B content (increased riboflavin content up to 5 mg/kg, increased folate content in the range of 150–340 μg/100 g), diminution of antinutrients and enhanced protein digestibility (10%−15%) (94). The complex carbohydrates and prebiotic fibers promote healthy microbial growth resulting in more short chain fatty acids and bioactive compounds. Mineral solubilisation is significantly improved with bioavailability of iron and zinc being up to two times higher following fermentation. Standardized biofortification targets for non-traditional substrates encompass a riboflavin increase to about 2.5–5 mg/kg, a folate increase of between 150 and 340 μg/100 g, a phytate decrease of up to 70% and mineral (Fe^2+^, Zn^2+^, or Ca^2+^) absorption increases of between 10 and 20% or more. Essential amino acids gain and protein digestion gain are spread all the way from 10 to 15%, depending on the protein contents of the substrate (26, 95).

## 6 Health implications and bio-efficacy

Microbes produce vitamins that have several health impacts on us as shown in Figure 3. These vitamins affect the micronutrient content, gut health and immunity. Studies in the lab, in animals and in humans demonstrate the role that microbe-made vitamins play in aiding body processes and preventing disease. They also demonstrate how these vitamins interact with our own flora to alter health outcomes. Cell culture models indicate how human cells absorb the vitamins produced by microbes, such as folate, riboflavin, vitamin B_12_ and vitamin K (2). For example, some lactic acid bacteria have the ability to produce folate which assists in the synthesis and repair of DNA in cultured mammalian cells. Riboflavin by microbes enhances mitochondrial energy production by being FAD and FMN, essential redox cofactors. Microbial vitamin B_12_, which is synthesized only by certain gut bacteria, is necessary for methylation reactions and blood cell production, which was confirmed when supplementation of cell cultures with B_12_ improved hematopoietic cell lines. Vitamin K in particular is the bacterial form of menaquinone, which activates the enzyme gamma-glutamyl carboxylase that is required in blood clotting and bone metabolism. From *in vitro* experiments, greater carboxylation activity was evident when cells were exposed to microbial vitamin K (96, 97).

Animal studies provide more understanding due to simulations of complex host-microbe interactions. Germfree mice colonized with the vitaminproducers had higher levels of vitamin as compared to germfree controls demonstrating that the microbes directly contribute to the body's storage of vitamins. Mice colonized with the folateproducing Bifidobacterium had more folate in the blood and tissue, and this increased the speed of growth in the mice and enhanced brain function. Mice that were colonized with the vitamin B_12_ producing bacteria Propionibacterium had greater haematocrit and fewer signs of anemia. Lactococcus strains producing riboflavin helped to increase antioxidant enzymes and reduce liver oxidative stress. Mice with bacteria which produced menaquinone-7 had a better calcium metabolism and bone density. These animals also displayed an improvement in immune balance and increased gut barriers, presumably due to immune cell cofactors such as microbial vitamins. However, the effects are not the same for all bacterial strains and microbial community, so not all microbes function in the same manner (98, 99).

These findings have been supported by human studies. Eating fermented foods rich in Vitamin LAB is associated with an improved status of micronutrients. One study found that consumption of probiotic drinks containing lactic acid bacillus capable of producing folate increased blood folate moderately and improved two markers of DNA methylation and cell division. Probiotics conferring a riboflavin production have been used to decrease the frequency of migraine, presumably by increasing mitochondrial energy generation (2, 100). Probiotic strains that produce vitamin B_12_ are being tested in order to correct B_12_ deficiency in the vegetarian population; preliminary trials indicate increased of B_12_ in blood and improved nerve function. Foods high in vitamin K_2_ or probiotics that produce vitamin K_2_ have been linked to increased bone density and reduced calcification of arteries comparable to the function of vitamin K in carboxylation of proteins. Metagenomic analyses reveal that different gut enterotypes have different vitamin synthesis capabilities, which are related to vitamin concentrations found in body fluids. Yet age, genetics, where one starts and the diet affect how people will respond, and therefore individuals respond differently (101, 102). Microbial vitamins are an important contribution to the body's pool of vitamins, and in some cases 10%−50% of the daily requirement. Folate produced by the gut helps produce blood cells, brain function and the fetal. Microbial riboflavin helps keep the cells in balance with oxidation and an aid in the use of energy. Endogenous vitamin B_12_ prevents pernicious anemia and relies on brain health. Vitamin K_2_ is present in the intestine, which regulates clotting as well as bone turnover. When these vitamins are in deficit because of poor absorption and/or low diet and a brokendown vitaminproducing microbiome, people get anemia, nerve issues, osteoporosis and immune problems (103).

The gut microbiota helps to keep the gut lining strong, they provide the cofactors required for the DNA building blocks and cell growth (104). Folate impacts proteins which seal the gut cells making them less leaky and inflammatory. Riboflavin reduces oxidative stress in the gut and that protects cells against the action of bad microbes (105, 106). Vitamin B_12_ promotes differentiation of gut cells and provides colon cell energy. Vitamin K_2_ maintains the immune balance and barrier functionality in the gut, a microbiome that manufactures lots of vitamins is likely to be diverse and resilient, less favorable to harmful bacteria and inflammatory bowel disease risk is decreased. Thus, vitamin production by microbes directly supports the health of the gut as well as the rest of the metabolism (107, 108). Microbemade vitamins then also work in concert within the microbiome. Some types of bacteria produce vitamins which others lack, so there is a network of shareable nutrients. This cross feeding makes the community stronger and diversity high. Vitamins affect the communication of the bacteria such as quorum sensing and biofilm formation and also how bacteria communicate with their host immune system. In the body, microbe-vitamins are responsible for modulating the immune responses, for example vitamin B complex influences the growth of T-cells in the body among others and vitamin K stimulates enzyme activation for blood clotting and bone remodeling. The total supply of dietary and bacterial vitamins influences the immune tone i.e., making the barriers stronger and fighting infections. When vitamin production is cut and dysbiosis takes place these partnerships break down, leading to increasing the risk of inflammatory and metabolic disease. Therefore, vitamins, microbiota and immune system are a connected network necessary for health and disease prevention (109–111).

## 7 Challenges and limitations

The use of vitamin producing microbial strains in food and pharmaceutical products has a number of critical challenges and limitations. These involve the viability and stability of strain during processing and storage, prevention of sensory changes that would deter consumers, and complex regulatory frameworks and standardization issues. One major issue is sustaining the viability and stability of the strains under processing, storage and distribution conditions. Microbial producers of vitamins such as folate, riboflavin, B_12_ and K are commonly used in fermented products or probiotic products. Industrial conditions like heat, pH adjustments, oxygen, moisture and levels of nutrients can minimize strain survival and metabolic activity. Strains with a known biosynthetic ability for vitamin formation, for example *Lactobacillus plantarum* or species of Bifidobacterium, can be at risk of loss in the drying processes used e.g., in spray drying or freeze drying, if protective agents and process parameters are not optimized. Even the process of refrigeration can reduce the number of viable cells over time, so that after a food is consumed there are less able to produce vitamins. This loss of viability diminishes the effectiveness of both fortification in terms of effectiveness and the health benefits usually provided by probiotics in terms of viability, since active and metabolically competent cells must be present in order for vitamin synthesis to take place (112–114). In addition to these traits, the stability of the vitamin synthesis ability of the strain recognizes that there is variation, with some strains being better than others determined by genetic robustness and cellular stress responses to stay genes expressed and enzymes functioning better when under stress. Therefore, formulations may include cryoprotectants and microencapsulation and carefully controlled storage situations which increase complexity and cost. Batch to batch variability of microbial populations also makes it more difficult to achieve consistent biofortification levels for consumers. Sensory changes as a result of microbial fermentation are also one reason for a limited acceptance of the biofortified products by consumers. At the same time that metabolic activities promote vitamin elevation, they also form organic acids, volatile compounds, and peptides that change taste, aroma, texture, and appearance. For example, build-up of lactic acid will render food more action of proteases liberates peptides that can be bitter, in taste. Vitamin-producing strains such as *Propionibacterium freudenreichii* can create increases in B_12_ content however generates propionic acid which produce cheesy or sharp off-flavors. Similarly, *Bacillus subtilis* natto is known to cause vitamin K_2_ increase in fermented soy, but is nasty in terms of ammonia likeodors which may finish off palatability. These sensory characteristics, however, may be off-putting, especially when frozen foods are introduced into the market where they are less familiar. Balancing high synthesis of vitamins with low sensory influence requires a balanced choice of strains, adequate fermentation control can one working certain formulation strategies such as flavor masking or blending with other food ingredients. Acceptance studies on consumer taste indicate it rises according to the cultural background, familiarity with fermented foods and individual taste preferences. Thus, sensory optimisation remains a very important, yet difficult, component of successful vitamin biofortification (112, 115).

Stability losses during storage defeat the nutrient claims of microbial biofortification in fermented foods. These losses bring about reduced amounts of bioavailable vitamins, probiotics, and bioactive metabolites such as short chain fatty acids (SCFAs), and invalidates the original enrichment data. For instance, viable numbers of Lactobacillus and bifidobacterium are reduced one to two log CFU/gram over 4 weeks at room temperature. The decrease includes developments resulting from acidification, oxygen and a phage activity, and the count falls below the probiotic threshold of 10^7^ CFU/g. Vitamins are also degraded during storage. Riboflavin (B_2_) and folate undergo a loss of 20%−40% of their content as a result of photo-oxidation and thermal lability, respectively. Provitamin A carotenoids in pearl millet ferments reduced by 30% after 28 days at 25 °C as compared with less than 15% loss when kept at refrigerated temperature. The efficacy reduction in *in vivo* rodent forms is proven (1, 116). Stored biofortificants elicit 25%−35% less caecal butyrate and have less shifts in clostridial, which are anti-inflammatory benefits reduced compared to fresh products. Regulatory claims must therefore incorporate a shelf life validated for stability, using methods such as using a highway to inspect the stability using, for example, the use of the techniques of the Human serum samples both techniques include monitoring of the stability of the product to verify that it provides the nutrients it claims to. Degradation destroys bioavailability and health results. Microencapsulation or lyophilization are means of decreasing such losses to as much as over 85% of activity, and support credible labeling (117).

Regulatory frameworks and standardization are also major challenges. Microbial vitamin biosynthesis and use of probiotics are covered by differing regulations in different jurisdictions, which impact on claims, labeling, safety evaluation, and manufacturing standards. Agencies like the US FDA, EFSA in Europe and others in the national authorities require the demonstration of clear evidence of safety, efficacy and quality control for using probiotic and fortified products. Vitamin production by microbes raises some unique concerns for the following reasons genetic and metabolic diversity of strains require a comprehensive characterization of biosynthetic gene clusters, demonstration of the absence of virulence factors or transferable antibiotic resistance and stability of functional characters (59, 112). Standardizing the vitamin content is challenging due to variability in strains, the dynamics of the fermentation process and the effect of the matrix, which in turn makes quality control challenging, as well as maintaining consistent dosing. Lack of harmonized standards for the labeling of microbial vitamin producers and their health claims are additional sources of regulatory complexity and can be confusing for the consumer. For example, the concentration range limits and methods for validating vitamin concentrations on a case-by-case basis vary depending on the country where products are sold, and legislations related to novel foods or supplements may put restriction on genetically modified or commonly novel microbiotabased products. Achieving regulatory approval requires comprehensive documentation of stakeholders such as strain identity, stability, bioavailability of vitamins produced and clinical proof of health benefits an extensive research and product development burden. Intellectual property issues, patents on the strains, etc., and proprietary fermentation processes also affect the access to the market and innovation. This fragmented regulatory landscape makes it hard for technologies for biofortification to be widely adopted commercially and makes scaling biofortification technologies more difficult around the world (113, 114).

## 8 Future directions

In microbial vitamin biosynthesis, the future of microbial vitamin biosynthesis is to make use of advances in genetic engineering, personalized nutrition and publichealth strategies. These advances will be supported by interdisciplinary collaboration, to overcome current limits; and to maximize actual impact. Engineered strains of probiotics are a promising solution to make more vitamin production. By adding or refining pathways to synthesize folate, riboflavin, vitamin B_12_ and vitamin K, scientists have been able to create “super – probiotic” strains which produce stable, strain-specific vitamins even when being industrially fermented and even within the gut. Technologies such as CRISPR-Cas-mediated genome editing, synthetic biology, pathway modularisation to insert delete control biosynthetic gene cluster This would mean a balance between the synthesis of the vitamins and the health and fitness of the strain (118). Engineered microbes can also be programmed to detect signals from the environment or the host and begin producing vitamins only when needed, thereby improving on bioavailability and reducing waste anyhow. By taking this genetic approach, problems of varying vitamin output and stability through processing are addressed, and is able to provide high yield probiotic products, both tailored for particular dietary or for therapeutic implementation. Integration of microbial vitamin biosynthesis with personalized nutrition and microbiome profiling is another way forward. Highresolution sequencing and metagenomic analysis to map their gut bacteria and its functional capacity for each person reveals the gaps in vitamin producing microbes that result in micronutrient shortfalls. Personalized diets or probiotic treatments can then be formulated to either restore or enhance those lost functions to improve their micronutrient status in accordance with a person's genetics, lifestyle and health profile. Advanced bioinformatics and machine learning predict the optimal microbial communities as well as vitamin dosages of the single individual. In addition, the probiotics can be combined with prebiotics and dietary fibers that selectively feed these microorganisms, making tailor-madesynbiotic formulations, which then can be used in addition. This precisionnutrition model promises to provide precision prevention and treatment of vitamin deficiencies and vitamin disorders, allowing for better efficacy without having to give vitamin supplementation unnecessarily (119, 120).

Benefits and challenges of microbial vitamin biosynthesis in low resource and public health settings growing microbial biofortification of vitamins in low resource and public health settings is a global priority. Micronutrient deficiencies impact millions of people, particularly those in underserved populations who are based on plant-based staples poor in vitamins, such as B_12_ and K. Development of low-cost and culturally acceptable fermented foods and probiotic products enriched by microbial vitamin manufacture may sustainably address these nutrition gaps. The use of locally grown fermented systems containing vitamin producing strains adapted to local conditions, is a cost-effective, accessible alternative to the synthetic supplements. Education and capacity building programmes empower the communities to use microbial biofortification for better nutrition (2). Microbial synthesis of vitamin can also be incorporated into emergency nutrition and maternal and child health interventions, lowering illness related to vitamin deficiency. Overcoming challenges in the areas of production, storage and taste in these contexts requires constant innovations and inclusive policies, which support technology transfer and regulatory harmonization. Realization of these future directions requires research across the boundaries of microbiology, genomics, food science, nutrition, clinical medicine and regulatory science. Collaboration is important for understanding microbe-hostnutrient interactions, building robust engineered strains, optimizing fermentation and conducting rigorous clinical potentiation and clinical trials for efficacy proof of concept. Systems biology and metabolic modeling will make the optimisation of strains more efficient. New delivery methods (e.g., microencapsulation) enhance the survival of probiotics and vitamin stability. Standards, safety checks, and consumer engagement have to keep pace with the level of product quality and acceptance. Public-privates and global consortia can be used to help accelerate the pathway from research to market and to public health impact. Holistic interventions that integrate the microbial production of vitamins with more general nutritional interventions may holistically address hidden hunger (114).

## 9 Conclusion

Microbial biofortification through the biosynthesis of vitamins by probiotics is a sustainable and natural approach for enhancing micronutrient nutritional status on a global scale. The microbial production of essential vitamins such as folate, riboflavin, vitamin B_12_ and vitamin K is an important part of the blend of enhanced nutrition that can be obtained from the widespread consumption of fermented foods which can provide fortified nutrient profiles without disrupting cultural food traditions. Advances in the understanding of metabolic pathways and strain-specific biosynthetic abilities is the reason for being able to acquire targeted vitamin increases in diverse foods. Research from *in vitro* to human findings has confirmed that the bio-efficacy of dietary microbe sources vitamins is important in improving micronutrient status, gut health and immune function. Nonetheless, challenges remain in ensuring strain stability and viability during processing and storage, managing sensory attributes to maximize consumer acceptability and navigating complex regulatory frameworks that influence standardization and product claims. Infuture highlight the possibility of advancing biosynthesis in probiotic strains by genetic engineering, personalized nutrition strategies by micropore profiling and implementation for low resources by socially acceptable fermented foods. These efforts require an interdisciplinary, collaborative approach that requires the interaction of microbiology, food technology and nutrition science as well as public health policy.
